# The mRNA Translation Inhibitor Vioprolide A Prevents Inflammatory Pain‐Like Behaviour With Limited Action on Already Established Pain‐Like Behaviour in Mice

**DOI:** 10.1002/ejp.70099

**Published:** 2025-08-13

**Authors:** Patrick Engel, Tilman Gross, Gesine Wack, Rekia Sinderwald, Luisa Burgers, Robert Fürst, Achim Schmidtko

**Affiliations:** ^1^ Institute of Pharmacology and Clinical Pharmacy, Goethe University Frankfurt Frankfurt am Main Germany; ^2^ Institute of Pharmaceutical Biology Goethe University Frankfurt Frankfurt am Main Germany; ^3^ Pharmaceutical Biology, Department of Pharmacy – Center for Drug Research Ludwig‐Maximilians‐Universität München Munich Germany

## Abstract

**Background:**

Accumulating evidence indicates that pharmacological inhibition of the translational machinery is a therapeutic strategy for various diseases. However, whether inhibitors of mRNA translation might be suitable for pain therapy remains poorly understood. Here, we tested the potential analgesic effects of the natural product vioprolide A, which targets nucleolar protein 14 (NOP14) that is essential for ribosome biogenesis, in mouse models of pain.

**Methods:**

We assessed the antinociceptive effects of vioprolide A in C57BL/6 mice using four different models: zymosan‐induced peritonitis, zymosan‐induced paw inflammation, complete Freund's adjuvant‐induced paw inflammation and spared nerve injury. Plasma and brain levels of vioprolide A were determined in a pharmacokinetic study. Immunostaining and western blot experiments were performed to investigate the distribution and expression of NOP14 in dorsal root ganglia.

**Results:**

Pretreatment with vioprolide A alleviated the visceral inflammatory hypersensitivity during zymosan‐induced peritonitis, and it attenuated the somatic inflammatory hypersensitivity during zymosan‐induced paw inflammation in a dose‐dependent manner. However, treatment with vioprolide A did not affect established hypersensitivities. Pharmacokinetic measurements revealed that vioprolide A was not brain‐penetrant and exhibited a short plasma half‐life, which however seems to be sufficient to exert long‐lasting antinociceptive effects. Tissue stainings revealed that NOP14 is expressed in a population of sensory neurons.

**Conclusions:**

Our findings imply that vioprolide A may alleviate inflammatory nociceptive behaviours, but highlight that these effects may be limited to specific types of pain and treatment strategies.

**Significance Statement:**

The inhibitor of mRNA translation, vioprolide A, produced robust antinociception in distinct murine models of pain. This study provides evidence supporting further investigation of mRNA translation inhibitors, which attenuate pain by a novel mechanism of action that is not shared by established analgesics.

## 1 Introduction

Chronic pain is a leading cause of disability worldwide and is associated with substantial personal and societal burden (Cohen et al. 2021; Fiore et al. 2023). Based on a mechanistic description, major forms of chronic pain include nociceptive, neuropathic and nociplastic pain (Kosek et al. 2016). In addition, chronic pain arising from visceral organs (i.e., visceral pain) may differ from that from the somatosensory system (Jayakar et al. 2021). Numerous animal studies suggested that persistent pain is associated with plastic and functional changes in the nociceptive system, which leads to sensitization and often outlasts the actual healing process of the injured or inflamed tissue (Gold and Gebhart 2010; Ji et al. 2018). However, the mechanisms underlying pain sensitization and its persistence are poorly understood.

A mechanism contributing to pain sensitization is the alteration of mRNA translation in the nociceptive system. Several studies revealed that translational mechanisms regulating protein synthesis may underlie phenotypic changes in the sensory nervous system. For example, local mRNA translation in primary afferent fibres through the mammalian target of rapamycin (mTOR) has been shown to regulate nociception, and local treatment with the mTOR inhibitor rapamycin attenuated persistent pain‐like behaviour in rodents (Geranton et al. 2009; Jimenez‐Diaz et al. 2008; Price et al. 2007). Other studies revealed that mRNA of the voltage‐gated sodium channel Nav1.8 is transported into axons after injury where it can be locally translated at sites of injury (Ruangsri et al. 2011; Thakor et al. 2009). Furthermore, it has been shown that cytoplasmic polyadenylation element binding protein (CPEB), a RNA‐binding molecule that regulates the translation of mRNA in peripheral axons, and alpha‐calmodulin‐dependent protein kinase II contribute to hyperalgesic priming (a form of neuroplasticity in nociceptors), which can be reversed by administration of translation inhibitors to the peripheral terminals of nociceptors (Araldi et al. 2018; Bogen et al. 2012; Ferrari, Bogen, Chu, and Levine 2013; Ferrari, Bogen, and Levine 2013). In these and other studies, several potential targets for inhibition of specific translation regulation signalling pathways have been identified (for review, see Khoutorsky and Price 2018; Megat and Price 2018). It seems therefore possible that pharmacological manipulation of translational mechanisms might reverse the phenotypic shifts that drive chronic pain, thereby creating a new strategy for pain therapy.

**FIGURE 1:**
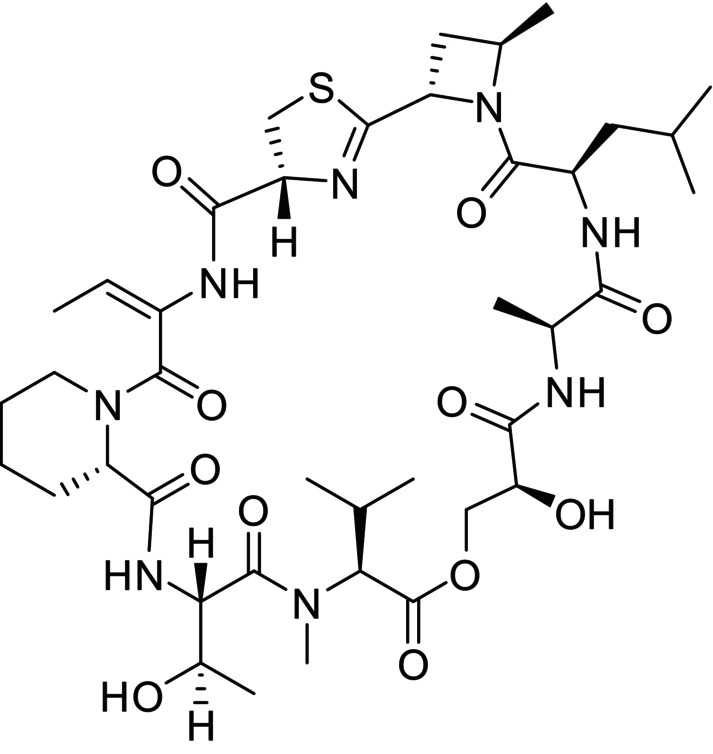
Structure of vioprolide A.

In a previous study we observed that treatment of mice with narciclasine, an inhibitor of translation elongation, significantly inhibited visceral pain behaviour in a murine peritonitis model (Stark et al. 2019). Similarly, we recently found that homoharringtonine, a compound that interferes with aminoacyl‐tRNAs at the 60S ribosomal subunit, ameliorated the pain behaviour during zymosan‐induced peritonitis (Burgers et al. 2024). Here, we tested whether antinociceptive effects are also achieved by vioprolide A, a peptolide isolated from *Cystobacter violaceus* (Figure 1; Yan et al. 2018). Vioprolide A is a translation inhibitor that targets the nucleolar protein NOP14, which is essential for ribosome maturation (Kirsch et al. 2020). Our data suggest that NOP14 is expressed in nociceptive sensory neurons and that administration of vioprolide A can attenuate visceral and somatic inflammatory pain in mice.

## 2 Methods

### 2.1 Animals

Experiments were performed with 8–12 week‐old male and female C57BL/6N mice purchased from Charles River (Sulzfeld, Germany). Animals were housed on a 12/12 h light/dark cycle with free access to water and food. All experiments were approved by the local animal welfare authorities (Regierungspräsidium Darmstadt, Germany; approval numbers V54‐19c20/15‐FR/1004 and V54‐19c18‐FR/2026), were conform to Directive 2010/63/EU, and adhered to the Animal Research: Reporting on In Vivo Experiments (ARRIVE) guidelines. All behavioural studies were conducted during the light cycle of the day by an observer blinded for the treatment of the animals. In the behaviour experiments, the treatment groups were randomised and evenly distributed across cages.

### 2.2 Zymosan‐Induced Peritonitis

Acute peritonitis was induced by a single intraperitoneal (i.p.) injection (0.5 mL) of a zymosan A suspension (2 mg/mL in phosphate‐buffered saline (PBS) without Ca^2+^/Mg^2+^; Sigma‐Aldrich, Steinheim, Germany and Gibco, Darmstadt, Germany) as described (Stark et al. 2019). Vioprolide A (0.3 mg/kg; kindly provided by the group of Prof. Rolf Müller, Helmholtz Institute for Pharmaceutical Research Saarland (HIPS), Helmholtz Center for Infection Research and Department of Pharmacy, Saarland University, Saarbrücken, Germany) or vehicle (1% DMSO in 0.9% NaCl) was subcutaneously (s.c.) injected into the neck (to prevent direct interaction with the i.p. applied zymosan) in a volume of 100 μL 16 h prior to the zymosan injection. An automated dynamic weight bearing (DWB) device (Bioseb, Boulogne, France) was used to assess the pain‐like behaviour during zymosan‐induced peritonitis. The device consists of an acrylic glass cage (11 × 11 cm) and an instrumented floor to measure the weight borne in each limb. A camera is installed above the cage to validate the animal positions during the recording. During a habituation phase of 5 min and a recording phase of 5 min, the mice were able to move freely in the cage. The DWB assessment was performed in naive mice (baseline) and 5 h after the i.p. zymosan injection. Following completion of each test, mice were removed, and the test chamber was cleaned with water. The recorded weight distribution was analysed using the DWB software as described (Laux‐Biehlmann et al. 2016; Stark et al. 2019). A zone was considered valid when the following parameters were detected: ≥ 0.8 g on one captor with a minimum of two adjacent captors recording ≥ 1.0 g. A time segment was considered valid if ≥ 3 stable pictures were detected (Laux‐Biehlmann et al. 2016; Stark et al. 2019). In all video sequences, the positions of the paws were manually validated by an observer. The mean weight over time was used to calculate the frontpaw to hindpaw ratio. Mice were excluded from the experiment if the frontpaw to hindpaw ratio at baseline was > 0.5.

### 2.3 Zymosan‐Induced Paw Inflammation

Paw inflammation was induced by injecting 20 μL of a zymosan A suspension (5 mg/mL in PBS without Ca^2+^/Mg^2+^, pH 7.4; Sigma‐Aldrich, St. Louis, USA) into the plantar side of a hindpaw (Petersen et al. 2019). Vioprolide A (0.3, 1 or 3 mg/kg) or vehicle (1% DMSO in 0.9% NaCl) was s.c. administered into the neck in a volume of 100 μL 16 h prior to the intraplantar zymosan injection.

Paw withdrawal latencies following mechanical stimulation were assessed using a dynamic plantar aesthesiometer (Ugo Basile, Gemonio, Italy), which compresses a stainless‐steel probe (0.5‐mm diameter) against the plantar surface of the hindpaw through a wire mesh bottom on which the mice are placed. The applied force was increased from 0 to 5 g over a period of 10 s (0.5 g/s ramp) and then remained constant at 5 g for an additional 10 s (total cutoff time, 20 s). An unequivocal paw withdrawal was evaluated as a response. The paw withdrawal latency was calculated as the average of three to four consecutive exposures with at least 20 s in between. The area under the curve was calculated using Prism 10 (Graph Pad) software.

The volume of the hindpaw was measured using a plethysmometer (Ugo Basile, Gemonio, Italy) according to the manufacturer's instructions. The dipping solution contained 0.8–0.9 g/L NaCl in distilled water and 2–3 mL/L wetting compound (Ugo Basile, Gemonio, Italy) to reduce drop and meniscus build‐up. The paw volume was calculated as a mean of three measurements. The area under the curve was calculated using Prism 10 (Graph Pad) software.

### 2.4 Complete Freund's Adjuvant (CFA)‐Induced Paw Inflammation

Paw inflammation was induced by injecting 20 μL of complete Freund's adjuvant (CFA; containing 1 mg/mL heat‐killed *Mycobacterium tuberculosis* in paraffin oil 85% and mannide monooleate 15%; Sigma‐Aldrich, St. Louis, USA) into the plantar side of a hindpaw (Petersen et al. 2019). Vioprolide A (0.3, 1 or 3 mg/kg), diclofenac (50 mg/kg; Sigma‐Aldrich, St. Louis, USA), or vehicle (1% DMSO in 0.9% NaCl) was i.p. administered in a volume of 100 μL 24 h after the intraplantar CFA injection. Paw withdrawal latencies following mechanical stimulation were assessed using a dynamic plantar aesthesiometer as described above.

### 2.5 Spared Nerve Injury (SNI)‐Induced Neuropathy

The SNI model was performed as described previously (Bourquin et al. 2006; Petersen et al. 2019). Briefly, under isoflurane anaesthesia the tibial and common peroneal branches of the sciatic nerve were ligated and dissected distally. The sural nerve remained intact and the mice developed hypersensitivity in the lateral area of the hindpaw. Vioprolide A (0.3 mg/kg) or vehicle (1% DMSO in 0.9% NaCl) were i.p. administered in a volume of 100 μL 14 days after SNI. Paw withdrawal latencies following mechanical stimulation were assessed using a dynamic plantar aesthesiometer as described above.

### 2.6 Accelerating Rotarod, Vertical Pole and Von Frey Filament Test

For the accelerating rotarod test, mice were placed on a rotarod treadmill (Ugo Basile, Gemonio, Italy) at increasing speed (4–40 rpm over 300 s; cutoff time 300 s) and trained for several consecutive days. Only mice that reached 300 s without falling off during the training sessions were included in the experiment. In the vertical pole test, mice were placed with the head pointing upward on a vertical pole and the time needed to reach the ground was recorded (cutoff time 20 s). After baseline measurements, vioprolide A (3 mg/kg) or vehicle (1% DMSO in 0.9% NaCl) was s.c. administered into the neck and the accelerating rotarod test immediately followed by the vertical pole test was performed 20 and 44 h after drug administration. Three trials per mouse were used for evaluation (Balzulat et al. 2024).

Immediately after the vertical pole test, mice were placed on a wire‐mesh platform in Plexiglas cylinders and habituated for approximately 1 h. Von Frey filaments ranging from 0.04 to 4 g (Ugo Basile, Gemonio, Italy) were applied to the plantar surface of a hind paw for 1 s with a force causing the filament to bend. Starting with the lowest force, each filament was applied 10 times with a break of at least 2 min. The number of paw withdrawal reactions per 10 applications was used to calculate the withdrawal frequency (Beaulieu‐Laroche et al. 2020). Measurements were performed at baseline and 22 and 46 h after vioprolide A or vehicle administration.

### 2.7 Pharmacokinetic Profile

The pharmacokinetic profile of vioprolide A was determined by staff of Pharmacelsus (Saarbrücken, Germany) in male C57BL/6 mice as described (Balzulat et al. 2024). A single dose of 0.3 mg/kg vioprolide A (in NaCl 0.9% + 1% DMSO) was i.p. administered, and two blood samples were obtained from each animal retrobulbarly under short isoflurane anaesthesia (at 0.25 and 0.5 h from 3 mice; at 1 and 2 h from 3 mice; and at 4 and 8 h from 3 mice). Immediately after the last blood sample was taken, the animal was euthanised by cervical dislocation, and the brain was collected. Plasma and brain concentrations of vioprolide A were determined by LC–MS analysis. The pharmacokinetic analysis was performed applying a non‐compartment model using the Kinetica 5.0 software (Thermo Scientific, Waltham, USA). All given parameters were obtained by trapezoid area calculation.

### 2.8 Tissue Staining

Mice were euthanized by CO_2_ inhalation and immediately perfused with 0.9% NaCl, followed by 1% paraformaldehyde (PFA) in PBS, pH 7.4. Dorsal root ganglia (DRGs) were dissected and cryoprotected in 30% sucrose in PBS overnight. Then tissues were embedded in tissue freezing medium (Tissue‐Tek O.C.T. Compound, #4583, Sakura, Torrance, CA) on dry ice, cryostat‐sectioned at a thickness of 14 μm on Superfrost Plus slides (# J1800AMNZ, Epredia, Braunschweig, Germany), dried at room temperature for 2 h and stored at −80°C until use.

In immunostaining experiments, slides were washed in PBS, permeabilised for 5 min in PBST (0.1% Triton X‐100 in PBS), blocked in 10% normal goat serum (#10000C, Thermo Fisher Scientific, Waltham, USA) plus 3% bovine serum albumin (BSA, #A6003, Sigma‐Aldrich, Darmstadt, Germany) in PBS for 1 h at room temperature, and then incubated with primary antibodies diluted in 3% BSA in PBS overnight at 4°C or for 2 h at room temperature. Primary antibodies included rabbit anti‐NOP14 (1: 1000; # PA5‐58851, Invitrogen, Waltham, USA), mouse anti‐neurofilament 200 (NF200; 1:1000; # N0142, Sigma‐Aldrich, Darmstadt, Germany), mouse anti‐peripherin (PRPH; 1:400; # MAB1527, Chemicon), rat anti‐F4/80 (1:500; # MCA497RT, Bio‐Rad, Feldkirchen, Germany), rat anti‐CD3 (1:500; # 14‐0032‐82, eBioscience, San Diego, USA) and mouse anti‐glutamine synthetase (GS; 1:500; GTX630654; GeneTex, Irvine, USA). In double‐labelling experiments, primary antibodies were consecutively incubated. Secondary antibodies conjugated to Alexa Fluor 488 or Alexa Fluor 555 (Thermo Fisher Scientific, Waltham, USA) were incubated in PBS at 1:1000 for 2 h at room temperature. After immunostaining, slices were treated with 0.06% Sudan black B (# 199664, Sigma‐Aldrich) in 70% ethanol for 5 min to reduce autofluorescence (Schnell et al. 1999), washed in PBS and mounted with Fluoromount G. In some tissue sections, the nuclei were stained with DAPI (600 nM; Invitrogen # D1306) for 15 min before mounting with Fluoromount G. Images were acquired using an Eclipse Ni‐U (Nikon, Düsseldorf, Germany) microscope equipped with a monochrome charge‐coupled device camera. The raw image files were brightened, contrasted, pseudocoloured and superimposed using ImageJ software (Schneider et al. 2012). Controls for immunostaining were performed by omitting the first and/or the second primary antibodies.

For the quantification of NOP14‐positive DRG neuron populations, serial sections of cervical (C3–C5), thoracic (T6–T12) and lumbar (L2–L4) DRGs from 7 mice were cut, and per animal, 2 sections at least 100 μm apart were counted manually by an observer. Only cells showing clear staining signals above the background level, with a threshold set based on incubation without primary antibody, were included.

### 2.9 Western Blots

Vioprolide A (0.3 mg/kg) or vehicle (1% DMSO in 0.9% NaCl) were s.c. administered into the neck in a volume of 100 μL. After 16 h, 20 μL of a zymosan A suspension (5 mg/mL in PBS without Ca^2+^/Mg^2+^, pH 7.4; Sigma‐Aldrich, St. Louis, USA) was injected into the plantar side of a hindpaw, whereas control animals (pretreated with vioprolide A or vehicle) did not receive a zymosan injection. After a further 24 h, mice were euthanized by CO_2_ inhalation and lumbar DRGs (L4–L5) were rapidly dissected, snap frozen in liquid nitrogen and stored at −70°C until use. Tissues were homogenised in RIPA lysis buffer supplemented with a protease inhibitor mixture (Protease Inhibitor Cocktail Tablets; Roche Diagnostics, Mannheim, Germany) and centrifuged for 30 min at 4°C and 13,000 rpm. Extracted proteins (30 μg/lane) were denatured with β‐mercaptoethanol (Rotiload; Carl Roth, Karlsruhe, Germany) and incubated at 95°C for 5 min. Then samples were separated by SDS‐PAGE (10%) and blotted onto a nitrocellulose membrane. After blocking of non‐specific binding sites with blocking buffer (Intercept Blocking Buffer; LI‐COR Biosciences, Lincoln, USA), membranes were incubated with rabbit anti‐NOP14 (1: 500; #PA5‐58851, Invitrogen, Waltham, USA) or mouse anti‐α‐tubulin (1:1000; #05‐829; Millipore, Billerica, USA) dissolved in blocking buffer containing 0.1% Tween 20 overnight at 4°C. After a washing step, secondary antibodies were incubated for 1 h at room temperature, and proteins were detected using an Odyssey Infrared Imaging system (LI‐COR Biotechnology, Bad Homburg, Germany). To quantify protein band intensity, the analysis tool of the Image Studio Lite software (LI‐COR Biotechnology, Bad Homburg, Germany) was used. All NOP14 levels were normalised relative to the level of α‐tubulin.

### 2.10 Statistical Analysis

**FIGURE 2:**
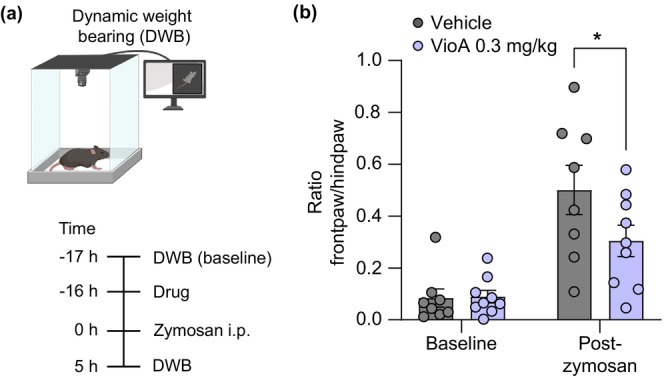
Vioprolide A reduces visceral nociceptive behaviour in a peritonitis model. (a) Illustration of the behavioural paradigm (top) and time course of the experiment (bottom). To assess the zymosan‐induced visceral nociceptive behaviour, the weight distribution changes of the front paws and hind paws were analysed using a dynamic weight bearing (DWB) device. After DWB baseline measurements, vioprolide A (VioA; 0.3 mg/kg) or vehicle (1% DMSO in 0.9% NaCl) were subcutaneously injected into the neck, and 16 h thereafter zymosan (1 mg) was intraperitoneally (i.p.) injected to induce peritonitis, followed by DWB assessment 5 h after the zymosan injection. (b) Summary graph showing that the extent of zymosan‐induced visceral nociceptive behaviour was significantly ameliorated by pretreatment with vioprolide A (*t*_(30)_ = 2.358, *p* = 0.0496, mixed‐effects analysis; *n* = 8–9, animals/group). Data are expressed as mean ± SEM. *p ≤ 0.05 vs. vehicle.

Statistical analysis was performed using Prism 10 (GraphPad). No statistical methods were used to pre‐determine the sample sizes; however, the sample sizes were similar to those reported in previous publications (Beckley et al. 2021; Laux‐Biehlmann et al. 2016; Stark et al. 2019) and standard practices in the field. A Kolmogorov–Smirnov test was used to assess the normal distribution of data within groups. Normally distributed data were analysed using one or two‐way repeated measures ANOVA or mixed‐effects analysis and are expressed as the mean ± standard error of the mean (SEM) or single data points with the mean ± SEM. Nonparametric data were analysed using a Kruskal–Wallis test and are presented as single data points with medians and interquartile ranges. One outlier (with ratio frontpaw/hindpaw > 8‐fold higher than the group mean of the vehicle group in the data shown in Figure 2) was excluded from the analysis in the entire study. For all statistical tests, a probability value of *p* < 0.05 was considered statistically significant. Asterisks in the figures indicate: ^#^, ^†^, *p < 0.05; **p < 0.01; ***, ^####^ *p* < 0.001; and ****p < 0.0001. Details of the analyses, including the statistical test, post hoc test and number of animals per group, are indicated in the figure legends.

## 3 Results

### 3.1 Vioprolide A Pretreatment Reduces Zymosan‐Evoked Visceral Nociceptive Behaviour

We first explored the antinociceptive effects of vioprolide A during zymosan‐induced peritonitis, a standard model of visceral pain. Mice were s.c. treated in the area of the neck with vioprolide A (0.3 mg/kg; the dose was selected based on a previous study (Burgers et al. 2021) and our own preliminary work) or vehicle, and 16 h thereafter the peritonitis was induced by i.p. injection of zymosan. The pain‐like behaviour was assessed using a dynamic weight bearing device before (baseline) and 5 h after the i.p. zymosan injection (Figure 2a). As expected, at baseline the mice put more weight on their hindpaws than on their frontpaws (Figure 2b). After the injection of zymosan, a weight shift towards the frontpaws was observed, indicating peritoneal inflammation. Interestingly, the zymosan‐induced weight shift was significantly lower in vioprolide A‐treated mice as compared to vehicle‐treated mice (Figure 2b). These data suggest that the translation inhibitor vioprolide A ameliorates visceral inflammatory hypersensitivity in mice.

### 3.2 Vioprolide A Pretreatment Reduces Zymosan‐Evoked Somatic Nociceptive Behaviour

**FIGURE 3:**
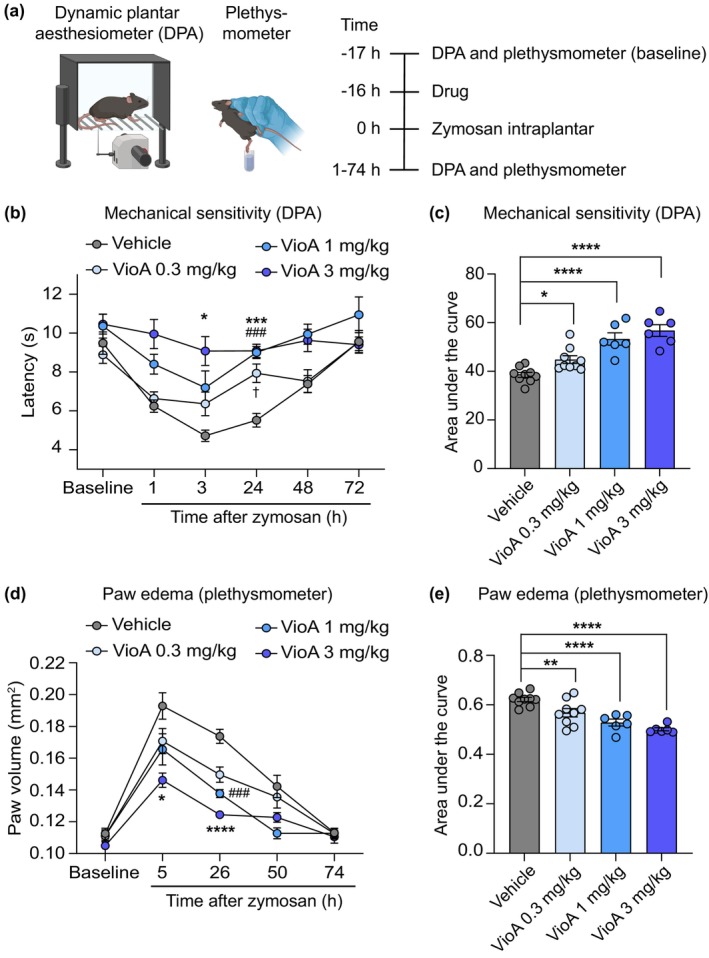
Vioprolide A reduces somatic inflammatory nociceptive behaviour and paw oedema. (a) Illustration of the behavioural paradigm (left) and time course of the experiment (right). To assess the zymosan‐induced somatic pain behaviour, the paw withdrawal latencies upon mechanical stimulation were determined using a dynamic plantar aesthesiometer (DPA). The paw volume was analysed using a plethysmometer. After baseline measurements, vioprolide A (VioA; 0.3, 1 or 3 mg/kg) or vehicle (1% DMSO in 0.9% NaCl) were subcutaneously injected into the neck, and 16 h thereafter zymosan (0.1 mg) was intraplantarly injected into a hindpaw. Further DPA and plethysmometer measurements were conducted during 1–74 h after the zymosan injection. (b, c) The zymosan‐induced mechanical hypersensitivity was significantly reduced after pretreatment with vioprolide A compared to vehicle in a dose‐dependent manner, as indicated in the time course of paw withdrawal latencies (b; *F*_3,26_ = 15.44, *p* < 0.0001; two way repeated measures ANOVA followed by Šidák's multiple comparisons tests; *p < 0.05, VioA 3 mg/kg vs. vehicle; ***p < 0.001, VioA 3 mg/kg vs. vehicle; ^###^ *p* < 0.001, VioA 1 mg/kg vs. vehicle; *n* = 6–9 animals/group) and the area under the curve (c; *F*_3,26_ = 20.55, *p* < 0.0001; ordinary one way ANOVA with Holm‐Šidák multiple comparison test; *p < 0.05, ****p < 0.0001 vs. vehicle; *n* = 6–9 animals/group). (d, e) The zymosan‐induced paw oedema was significantly reduced after pretreatment with vioprolide A compared to vehicle in a dose‐dependent manner, as indicated in the time course of paw volume (d; *F*_3,26_ = 16.51, *p* < 0.0001; two‐way repeated measures ANOVA with Šidák's multiple comparisons test; *p < 0.05, VioA 3 mg/kg vs. vehicle; ****p < 0.0001, VioA 3 mg/kg vs. vehicle; ^###^ *p* < 0.001, VioA 1 mg/kg vs. vehicle; *n* = 6–9 animals/group) and the area under the curve (e; *F*_3,26_ = 15.91, *p* < 0.0001; ordinary one way ANOVA with Holm‐Šidák multiple comparison test; **p < 0.01, ****p < 0.0001 vs. vehicle; *n* = 6–9 animals/group). Data are expressed as mean ± SEM.

We next assessed whether pretreatment with vioprolide A can ameliorate the mechanical hypersensitivity in a model of zymosan‐induced paw inflammation. Animals were s.c. administered in the area of the neck with different doses of vioprolide A (0.3, 1 and 3 mg/kg) or vehicle, and 16 h thereafter zymosan was intraplantarly injected into a hindpaw. The mechanical sensitivity of the hindpaws was assessed using a dynamic plantar aesthesiometer before (baseline) and during 1–72 h post‐zymosan. Furthermore, the extent of the paw edema was analysed using a plethysmometer (Figure 3a). Interestingly, after pretreatment with vioprolide A, the zymosan‐induced mechanical hypersensitivity was significantly ameliorated in a dose‐dependent manner as compared to vehicle control (Figure 3b,c). Furthermore, the extent of zymosan‐induced paw edema decreased in a dose‐dependent manner after pretreatment with vioprolide A (Figure 3d,e). Together, these data suggest that pretreatment with vioprolide A ameliorates the somatic inflammatory nociceptive behaviour and paw edema induced by zymosan.

In control experiments, we tested in naive mice (without intraplantar zymosan injection) the motor function and mechanical sensitivity during 20–46 h after administration of vioprolide A, that is, at time points of antinociceptive effects in the zymosan‐induced paw inflammation model. Treatment with vioprolide A (3 mg/kg s.c. into the neck) did not impair motor function in the accelerating rotarod test, a standard model of motor performance (Figure S1a), or in the vertical pole test, which assesses basal ganglia‐related movement disorders (Figure S1b). Furthermore, vioprolide A did not alter mechanical sensitivity, as analysed by stimulation of a hindpaw with von Frey filaments of increasing mechanical force (Figure S1c). These data suggest that motor function and mechanical sensitivity are not impaired after treatment with vioprolide A.

### 3.3 Vioprolide A Does Not Affect Persisting Inflammatory or Neuropathic Hypersensitivity

**FIGURE 4:**
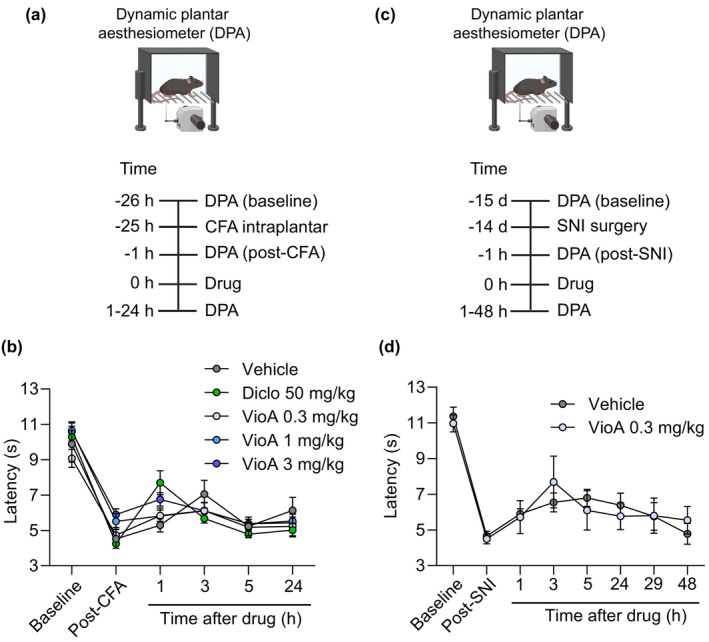
Persisting mechanical hypersensitivity is not affected by vioprolide A. (a, b) CFA‐induced mechanical hypersensitivity. (a) Illustration of the behavioural paradigm (top) and time course (bottom). Mice were injected with 20 μL complete Freund's adjuvant (CFA) into a hindpaw. Twenty‐four hours thereafter, a mechanical hypersensitivity of the affected hindpaw (determined using a dynamic plantar aesthesiometer) was detected in all mice. Then animals were i.p. treated with vioprolide A (VioA; 0.3, 1 or 3 mg/kg; *n* = 6 per group), 50 mg/kg diclofenac (*n* = 6) or vehicle (*n* = 6), and the mechanical sensitivity was assessed over 24 h. (b) The time course of paw withdrawal latencies shows that none of the applied doses of vioprolide A affected the CFA‐induced mechanical hypersensitivity (*F*_4,25_ = 1.282, *p* = 0.3036, two‐way repeated measures ANOVA). (c, d) Spared nerve injury (SNI)‐induced mechanical hypersensitivity. (c) Illustration of the behavioural paradigm (top) and time course (bottom). In the SNI model, neuropathic pain was induced by surgery. Fourteen days thereafter, a mechanical hypersensitivity of the affected hindpaw was detected in all mice. Then animals were i.p. treated with 0.3 mg/kg vioprolide A (*n* = 6) or vehicle (*n* = 5), and the mechanical sensitivity was assessed over 48 h. (d) The time course of paw withdrawal latencies shows that vioprolide did not affect the SNI‐induced mechanical hypersensitivity (*F*_1,9_ = 0.0002282, *p* = 0.9983, two‐way repeated measures ANOVA). Data are expressed as mean ± SEM.

Having established that pretreatment with vioprolide A can attenuate the nociceptive behaviour, we next investigated whether an already persisting hypersensitivity is affected by treatment with the translation inhibitor. To explore the effects of vioprolide A on established inflammatory hypersensitivity, we used a model of CFA‐induced paw inflammation, in which mechanical hypersensitivity persists longer than that in the zymosan model (Petersen et al. 2019). Vioprolide A at three doses (0.3, 1 and 3 mg/kg), diclofenac (50 mg/kg; used as a positive control), or vehicle were i.p. administered 24 h after the intraplantar CFA injection, that is, at a time point at which the mechanical hypersensitivity of the injected hindpaw had fully developed; thereafter, the mechanical sensitivity was assessed over 24 h (Figure 4a). As expected, the CFA‐induced mechanical hypersensitivity was visibly attenuated in the first hour after treatment with diclofenac (Figure 4b), albeit statistical significance was not reached. However, vioprolide A at all three doses failed to affect the mechanical hypersensitivity during the 24 h observation period (Figure 4b), suggesting that persisting inflammatory nociceptive behaviour is not affected.

We then evaluated the effects of vioprolide A on persisting neuropathic hypersensitivity. For this purpose, we employed the spared nerve injury (SNI) model of neuropathy induced by peripheral nerve injury using an experimental setting, in which we in a previous study observed that the positive control pregabalin attenuated the neuropathic pain behaviour (Metzner et al. 2021). Vioprolide A (0.3 mg/kg) or vehicle were i.p. administered on Day 14 after the SNI surgery (i.e., after the mechanical hypersensitivity of the affected hindpaw had fully developed), and the mechanical sensitivity was assessed over 48 h (Figure 4c). However, vioprolide A failed to affect the mechanical hypersensitivity in the SNI model (Figure 4d), similar to the CFA model. Together, these data suggest that persisting mechanical hypersensitivity is not affected by vioprolide A.

### 3.4 Pharmacokinetic Properties of Vioprolide A

We then investigated the pharmacokinetic properties of vioprolide A. After i.p. administration of 0.3 mg/kg vioprolide A in mice, plasma and brain levels were determined over 8 h using LC–MS. We found that vioprolide A concentrations in the plasma decayed with first‐order kinetics, with a half‐life of ~1.0 h (Table 1). Furthermore, vioprolide A was not detected in the brain, suggesting that it did virtually not cross the blood–brain barrier. Collectively, these data indicate that vioprolide A has a relatively short half‐life in the plasma, although its antinociceptive effects were seen at much later time points (as shown in Figures 2 and 3).

**TABLE 1:** Pharmacokinetic profile of vioprolide A. Mice were i.p. injected with 0.3 mg/kg vioprolide A and plasma and brain levels were measured by LC–MS analysis.

*C*_max_ (ng/mL)	7.8
*t*_max_ (h)	0.25
*C*_z_ (ng/mL)	0.1
*t*_z_ (h)	4.0
*t*_1/2z_ (h)	1.0
AUC_0‐tz_ (ng h/mL)	3.6
AUC_0‐inf_ (ng h/mL)	3.8
%AUC_extra_	3.9
*V*_z_/*f* (mL/kg)	115,926
CL/f (mL/(h kg))	79,561

*Note:* Shown are the calculated pharmacokinetic parameters in the plasma. The compound was not detected in the brain. *C*_max_, maximal concentration; *t*_max_, time to reach the maximum concentration; *C*_z_, last analytically quantifiable concentration; *t*_z_, time of the last sample which has an analytically quantifiable concentration; *t*_1/2z_, half‐life of the terminal slope of a concentration–time curve; AUC_0–tz_, area under the concentration–time curve up to the time *t*_z_ of the last sample; AUC_0‐inf_, area under the concentration–time curve extrapolated to infinity; %AUC_extra_, extrapolated part of AUC_0‐inf_; *V*_z_/*f*, volume of distribution after extravascular administration; CL/f, total body clearance after extravascular administration.

### 3.5 The Vioprolide A Target NOP14 Is Expressed in Nociceptive Sensory Neurons

**FIGURE 5:**
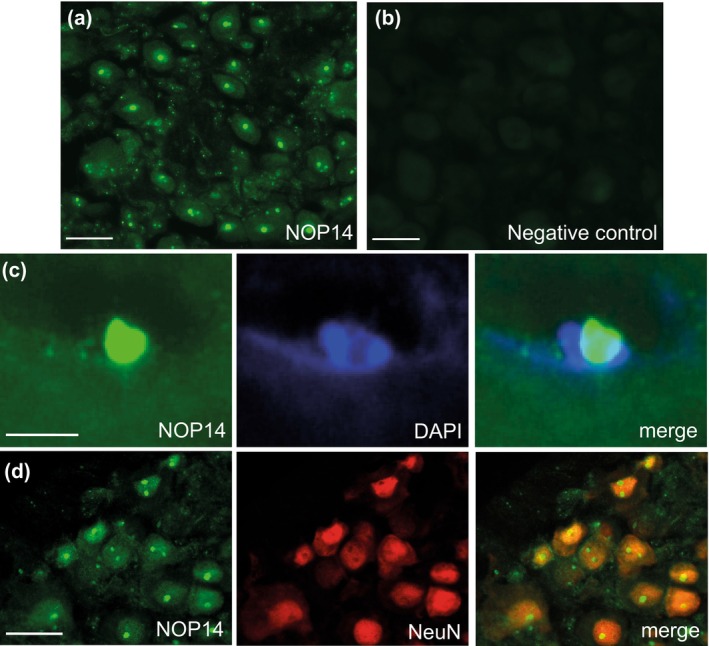
Distribution of the vioprolide A target NOP14 in dorsal root ganglia. (a) Immunostaining of NOP14 in lumbar dorsal root ganglia. (b) No immunoreactivity was observed by omitting the primary antibody. (c) Doublestaining of NOP14 and DAPI in DRG neurons revealed a nuclear localisation of NOP14. (d) Double immunostaining of NOP14 and NeuN demonstrates that NOP14 is expressed in neuronal nuclei. Scale bars: (a, b and d) 25 μm, (c) 5 μm.

**FIGURE 6:**
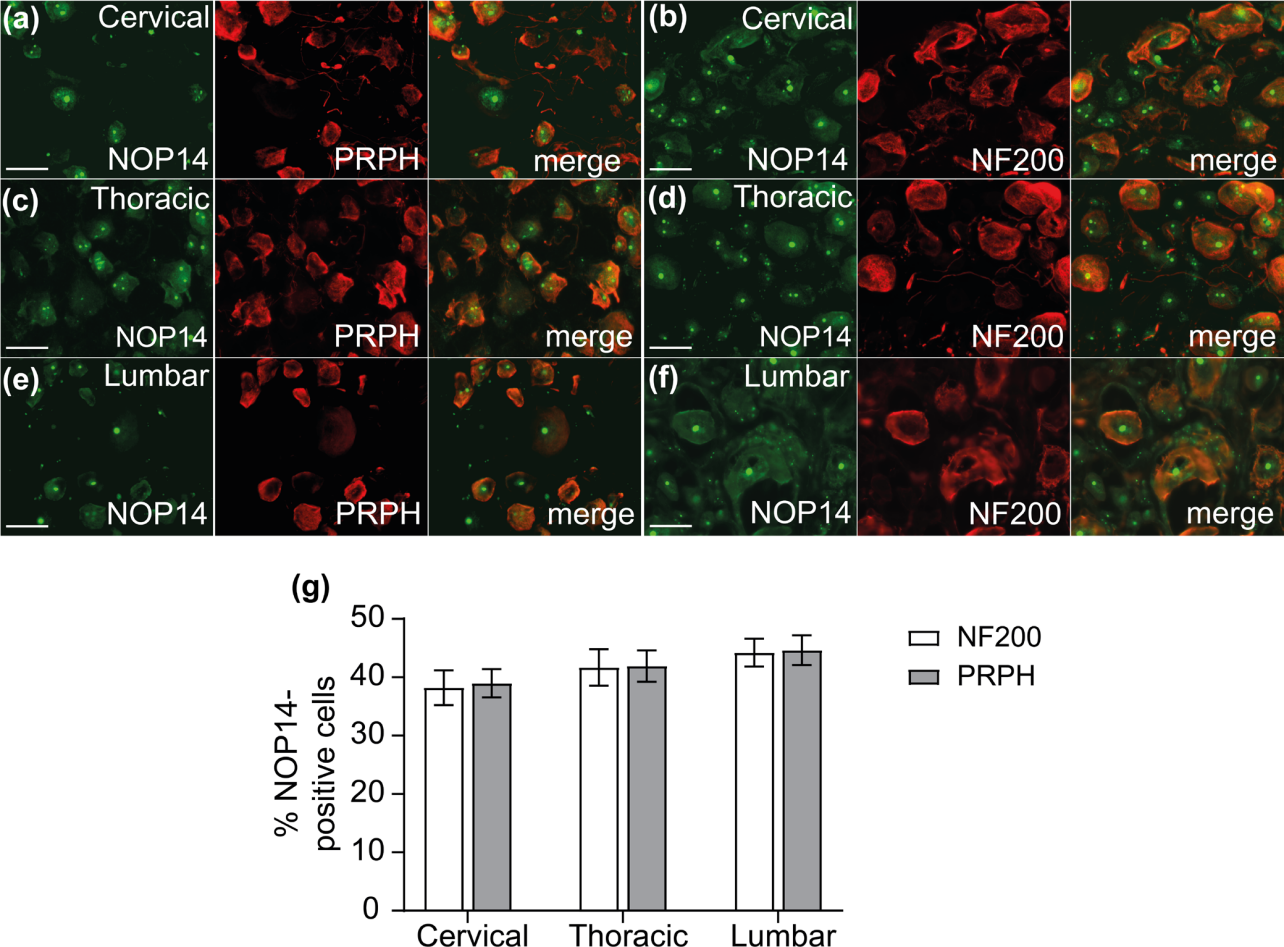
Distribution of NOP14 in neuronal cells in cervical, thoracic and lumbar dorsal root ganglia. (a–f) Double‐immunostaining of NOP14 with peripherin (PRPH), a marker of unmyelinated C‐fibre neurons (a, c and e) and neurofilament 200 (NF200), a marker of myelinated neurons (b, d and f) were performed on different segmental locations of the DRGs. (g) Percentages of PRPH‐positive or NF200‐positive neurons that co‐express NOP14 in cervical (4858 neurons counted; *n* = 7 mice), thoracic (4932 neurons counted; *n* = 7 mice), or lumbar (5733 neurons counted; *n* = 7 mice) DRG neurons. Data are expressed as mean ± SEM. Scale bars: 25 μm.

Finally, we investigated the cellular distribution of the vioprolide A target, NOP14, in dorsal root ganglia. In immunostainings, we found NOP14 immunoreactivity to be enriched in distinct small areas of neuronal somata (Figure 5a). No immunoreactivity was detected in control experiments without the primary antibody (Figure 5b). Costaining of NOP14 with DAPI (Figure 5c) and the neuronal marker NeuN (Figure 5d) revealed that NOP14 is expressed in DRG neurons and localised to the nucleus, corresponding to its expected function as a nucleolar protein. In further double‐immunostaining experiments, we assessed the expression of NOP14 in DRG neurons positive for peripherin (PRPH; marker of unmyelinated C‐fibre neurons) and neurofilament 200 (NF200; marker of myelinated neurons). These stainings were performed on cervical, thoracic and lumbar DRGs to analyse whether NOP14 expression differs depending on the segmental location of the DRG, as previously reported for TRP channel genes (Vandewauw et al. 2013). In cervical DRGs, NOP14 was expressed in 39.0% ± 1.2% of PRPH‐positive and 38.0% ± 1.5% of NF200‐positive neurons (Figure 6a,b,g). Similar distribution patterns were found in other segmental locations of the DRGs, with NOP14 being expressed in 41.9% ± 1.3% (thoracic) and 44.6% ± 1.3% (lumbar) of PRPH‐immunoreactive DRG neurons and in 41.7% ± 1.5% (thoracic) and 44.2% ± 1.2% (lumbar) of NF200‐immunoreactive neurons (Figure 6c–g). Taken together, these data suggest that the vioprolide A target, NOP14, is expressed in subpopulations of unmyelinated C‐fibre neurons and myelinated DRG neurons.

**FIGURE 7:**
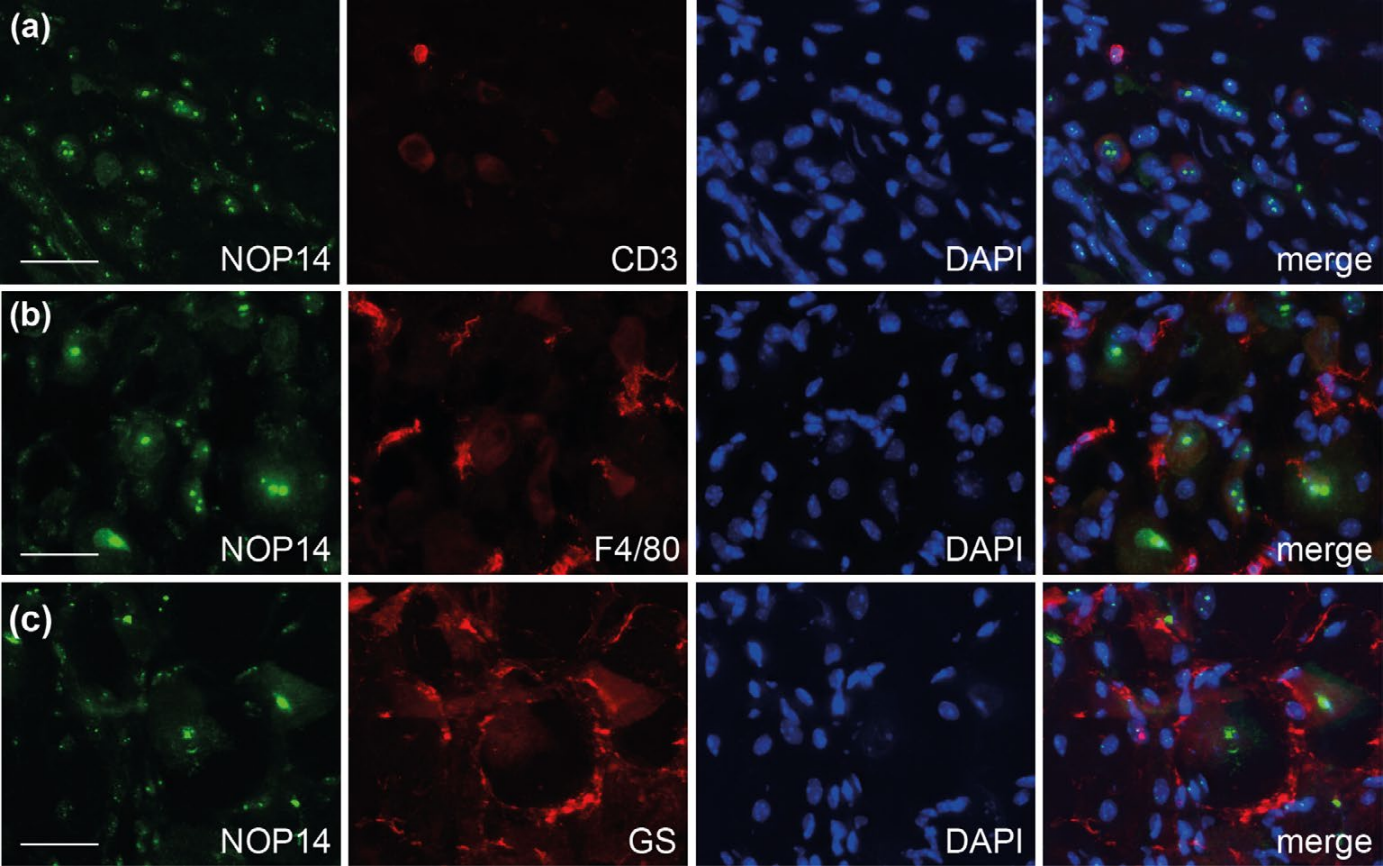
Distribution of NOP14 in non‐neuronal cells in lumbar dorsal root ganglia. Double‐immunostaining of NOP14 with CD3, a marker of T cells (a), F4/80, a marker of macrophages (b) and glutamine synthetase (GS; c), a marker of satellite glial cells. Data indicate that NOP14 is localised to a fraction of CD3‐positive T cells, but not F4/80‐positive macrophages or GS‐positive satellite glial cells. Representative stainings from *n* = 5 mice. Scale bars: 25 μm.

We performed further double‐immunostaining experiments in lumbar DRGs to estimate the expression of NOP14 in non‐neuronal cells and detected NOP14 immunoreactivity in a fraction of cells positive for CD3, a marker for T cells (Figure 7a). However, NOP14 immunoreactivity was not detected in cells positive for F4/80, which stains macrophages (Figure 7b), nor in cells positive for glutamine synthetase, which labels satellite glial cells (Figure 7c).

We then investigated whether vioprolide A treatment or a proinflammatory stimulus affects the expression of NOP14 in DRGs. In a setting similar to that of the zymosan‐induced paw inflammation model (Figure 3), vioprolide A (0.3 mg/kg) or vehicle was s.c. injected in the neck area; 16 h thereafter, animals received an intraplantar injection of zymosan into a hindpaw while control mice did not receive a zymosan injection, and after a further 24 h, animals were killed and L4–L5 DRGs were excised. Western blot analyses showed no change in NOP14 protein expression in the DRGs after vioprolide A treatment and/or zymosan‐induced paw inflammation (Figure S2). These data suggest that NOP14 expression in DRGs is not regulated after systemic administration of vioprolide A or intraplantar injection of zymosan.

## 4 Discussion

The research presented here demonstrates that inflammatory nociceptive behaviour in mice can be attenuated by a small molecule inhibitor of the translational machinery. However, our data also indicate that this compound inhibited the nociceptive behaviour only when administered before a proinflammatory stimulus was given in the pain models.

The translation of genetic information from mRNA into protein is a vital process that has been highly conserved throughout evolution. Alterations in the regulation of mRNA translation are increasingly recognised as an important mechanism in the pathophysiology of many diseases and disorders, including autoimmunity, diabetes, cancer, neurodegeneration and pain (Yousuf et al. 2021). Our finding that inflammatory nociceptive behaviour in mice was attenuated by the translation inhibitor vioprolide A is in line with our previous studies, in which the translation inhibitors narciclasine and homoharringtonine ameliorated the nociceptive behaviour in the zymosan‐induced peritonitis model in mice (Burgers et al. 2024; Stark et al. 2019). Vioprolide A, a peptolide isolated from *Cystobacter violaceus* in 1996 (Yan et al. 2018), inhibits NOP14, which is crucial for 40S ribosome subunit formation and maturation of 18S rRNA (Kirsch et al. 2020; Liu and Thiele 2001). Narciclasine is a phenanthridone‐type alkaloid isolated from the bulbs of *Narcissus* species in 1967 and inhibits peptide elongation by binding to the 60 S tRNA A‐site (Furst 2016). Homoharringtonine is an alkaloid from *Cephalotaxus* species that binds to the eukaryotic 60S large ribosomal subunit, thereby competing with the amino acid side chains of incoming aminoacyl‐tRNAs for binding in the A‐site cleft in the peptidyl‐transferase centre (Gurel et al. 2009). The observation that these three compounds with different molecular targets within the eukaryotic translational machinery attenuated the nociceptive behaviour induced by zymosan adds further evidence that inhibition of mRNA translation is a novel strategy for analgesia. However, it is important to note that the compounds were administered 12–16 h prior to the zymosan injection in these studies. By contrast, vioprolide A failed to affect the mechanical hypersensitivity when administered 1 day after intraplantar CFA injection or 14 days after SNI, respectively. This finding, which resembles the usual clinical setting more than a pretreatment before pain induction, limits the general transferability of the observed effects. The lack of effect on established pain‐like behaviours suggests that the timing of administration is critical and that vioprolide A might be more effective in preventing the onset of persistent hypersensitivity rather than reversing it once it has already established.

The finding that vioprolide prevented hypersensitivity but did not affect established hypersensitivity might also be related to mRNA translation mechanisms that differ before and after the pain conditions are installed. Before the onset of persistent pain, translational control in nociceptive neurons governs acute responses to noxious cues through a variety of mechanisms. It can be modulated by activation of various membrane receptors, such as tyrosine receptor kinases (TrkA and TrkB), metabotropic glutamate receptors, NMDA receptors and insulin‐like receptors (Khoutorsky and Price 2018), which, however, are distinct with respect to functional effects on translation (induction or repression). For example, the primary ligand for the TrkA receptor, nerve growth factor (NGF), is a potent activator of signalling through the mammalian/mechanistic target of rapamycin (mTOR) pathway in sensory neurons (Melemedjian et al. 2010). It has been shown that mTOR signalling regulates the rate of mRNA translation via phosphorylation of 4E‐binding protein, which promotes the assembly of the eIF4F complex, ribosome recruitment to the mRNA and initiation of translation of ‘eiF4E‐sensitive’ mRNAs (Khoutorsky et al. 2015; Thoreen et al. 2012). Another mTOR effector in sensory neurons is ribosomal protein S6 kinase 1 (S6K1), which mediates translation of c‐Fos and modulates the rapid response to inflammatory mediators (de la Pena et al. 2021). Furthermore, it has been recently found that metabotropic glutamate receptor 5 regulates pain signalling in sensory neurons through activation of elongation factor 2 kinase (eEF2K), which is capable of inducing the integrated stress response through ribosome‐dependent activation of the eIF2α kinase, GCN2, thereby suppressing global protein synthesis, but enabling the preferential translation of brain‐derived neurotrophic factor (BDNF) (Smith et al. 2025). Other mechanisms regulating translation initiation include phosphorylation of eukaryotic initiation factor 2α (Barragan‐Iglesias et al. 2019; de la Pena et al. 2023; Khoutorsky et al. 2016; Yousuf et al. 2023), regulation of the length of the poly(A) tail of mRNA (Barragan‐Iglesias et al. 2018; Bogen et al. 2012; Ferrari, Bogen, Chu, and Levine 2013) or microRNAs‐mediated effects (Zhao et al. 2010). During persistent pain, translational regulation shifts significantly. For example, in a mouse model of paclitaxel‐induced neuropathic pain, translating ribosome affinity purification (TRAP) sequencing revealed 160 genes (79 upregulated and 81 downregulated) with persistently altered mRNA translation in NaV1.8‐positive nociceptors (Sankaranarayanan et al. 2025), and ribosome profiling indicated that mRNA translation of 404 genes (371 increased and 33 decreased) was altered in DRGs (de la Pena et al. 2025) after the paclitaxel treatment. Moreover, it seems possible that these changes are not limited to cell bodies: increased local translation in axons has been suggested to support production of proteins at injury sites, thereby contributing to nociceptive signalling (Khoutorsky and Price 2018). However, the functional significance of local translation in axons during persistent pain remains poorly understood. Our observation that the vioprolide target NOP14 is localised to the nucleus of DRG neurons (Figure 5), corresponding to its anticipated function in nucleolar processing of pre‐18S ribosomal RNA and nuclear export of 40S pre‐ribosomal subunit to the cytoplasm (Dragon et al. 2002; Milkereit et al. 2003), supports the hypothesis that vioprolide A could mainly affect somatic protein synthesis rather than protein synthesis in axons. On the other hand, it cannot be excluded that an inhibition of ribosome biogenesis might manifest with deficits in both somatic and axonal protein synthesis. Further studies are required to elucidate how vioprolide A affects mRNA translation mechanisms that are important under conditions before persistent pain has developed.

Despite the promising results, there are several limitations to this study. One major limitation is that the study focused on specific models of pain in mice, and it is unclear whether these findings can be generalised to other types of pain or to human subjects. Other limitations are that we do not provide evidence on a cellular level that vioprolide A impedes translation in sensory neurons, modulates NOP14 functions in sensory neurons, or causes loss of ribosome maturation in vivo. Furthermore, about the mechanisms that underlie the attenuated nociceptive behaviour after pretreatment with vioprolide A we can only speculate. Considering the pharmacokinetic data, which indicate that vioprolide A has only a short plasma half‐life, it seems possible that the production of proinflammatory and pronociceptive proteins might be inhibited by vioprolide A and that this mitigates subsequent signalling cascades. In fact, our earlier studies revealed that narciclasine and homoharringtonine reduced the amount of cytokines including interleukin‐1β, interleukin‐6 and tumour necrosis factor in the peritoneal lavage after zymosan‐induced peritonitis (Burgers et al. 2024; Stark et al. 2019), suggesting an attenuated inflammatory response to the zymosan injection. The finding that vioprolide A, in a dose‐dependent manner, reduced the extent of paw oedema after the intraplantar zymosan injection is in line with this assumption, and the attenuated paw inflammation could have contributed to the inhibition of pain‐like behaviour. Nevertheless, further studies are needed to unravel the mechanism by which translation inhibitors exert antinociceptive effects.

## 5 Conclusion

In the current study, we report for the first time that vioprolide A exerts antihyperalgesic effects in mice. Our data add further evidence that inhibitors of the translational machinery may be promising novel analgesics. Future research addressing the mechanistic basis of the antinociceptive effects could have profound effects on pain modalities implemented clinically.

## Author Contributions

Patrick Engel performed experiments, analysed and interpreted data, prepared figures and contributed to the writing of the article. Tilman Gross performed experiments, analysed and interpreted data, prepared figures and contributed to the writing of the article. Gesine Wack analysed data. Luisa Burgers performed experiments. Rekia Sinderwald performed experiments. Robert Fürst acquired funding for the study, contributed to the study design and critically revised the article. Achim Schmidtko acquired funding for the study, designed the study, supervised the behavioural and tissue staining experiments and wrote the article. All authors discussed the results and commented on the manuscript.

## Conflicts of Interest

The authors declare no conflicts of interest.

## Supporting information


**Figure S1:** Vioprolide A does not impair motor function and mechanical sensitivity. (a and b) Motor function. Vioprolide A (VioA; 3 mg/kg) or vehicle (1% DMSO in 0.9% NaCl) were subcutaneously (s.c.) administered into the neck area, and an accelerating rotarod test (a) followed by a vertical pole test (b) was performed 20 and 44 h thereafter. Data show that vioprolide A did not affect the time spent on the rotarod or the vertical pole. Box‐and‐whisker plots represent maximum and minimum values, and the box shows the first, second (median), and third quartile values. Dotted lines indicate the maximum test time (cutoff time). *n* = 8 mice per group. (c) Percentage of paw withdrawals in response to von Frey filaments at different forces (10 trials per filament) in mice 22 and 46 h after s.c. administration of vioprolide A or vehicle. BL, baseline. Data are means ± SEM from *n* = 8 mice per group.

**Figure S2:** NOP14 expression in dorsal root ganglia is not regulated by vioprolide A or zymosan. Sixteen hours after s.c. administration of vioprolide A (VioA; 0.3 mg/kg) or vehicle, mice received an intraplantar injection of zymosan (zym) into a hindpaw while control mice did not receive a zymosan injection. After 24 h animals were killed and L4–L5 DRGs were excised. (a) Representative Western blot of NOP14 (calculated molecular weight: 98 kDa) with DRG homogenates. α‐Tubulin (52 kDa) was used as loading control. (b) Densitometric analyses did not detect altered expression of NOP14 after vioprolide A treatment and/or intraplantar zymosan injection. *n* = 3 animals per group.
