# Supplementary material for: The mRNA Translation Inhibitor Vioprolide A Prevents Inflammatory Pain‐Like Behaviour With Limited Action on Already Established Pain‐Like Behaviour in Mice
Source: Eur J Pain. 2025 Aug 13;29(8):e70099. doi: 10.1002/ejp.70099 (PMC12345401; doi:10.1002/ejp.70099)
Supplement: Supplementary file 1 — Figure S1: Vioprolide A does not impair motor function and mechanical sensitivity. (a and b) Motor function. Vioprolide A (VioA; 3 mg/kg) or vehicle (1% DMSO in 0.9% NaCl) were subcutaneously (s.c.) administered into the neck area, and an accelerating rotarod test (a) followed by a vertical pole test (b) was performed 20 and 44 h thereafter. Data show that vioprolide A did not affect the time spent on the rotarod or the vertical pole. Box‐and‐whisker plots represent maximum and minimum values, and the box shows the first, second (median), and third quartile values. Dotted lines indicate the maximum test time (cutoff time). n = 8 mice per group. (c) Percentage of paw withdrawals in response to von Frey filaments at different forces (10 trials per filament) in mice 22 and 46 h after s.c. administration of vioprolide A or vehicle. BL, baseline. Data are means ± SEM from n = 8 mice per group. [file EJP-29-0-s001.docx]

**
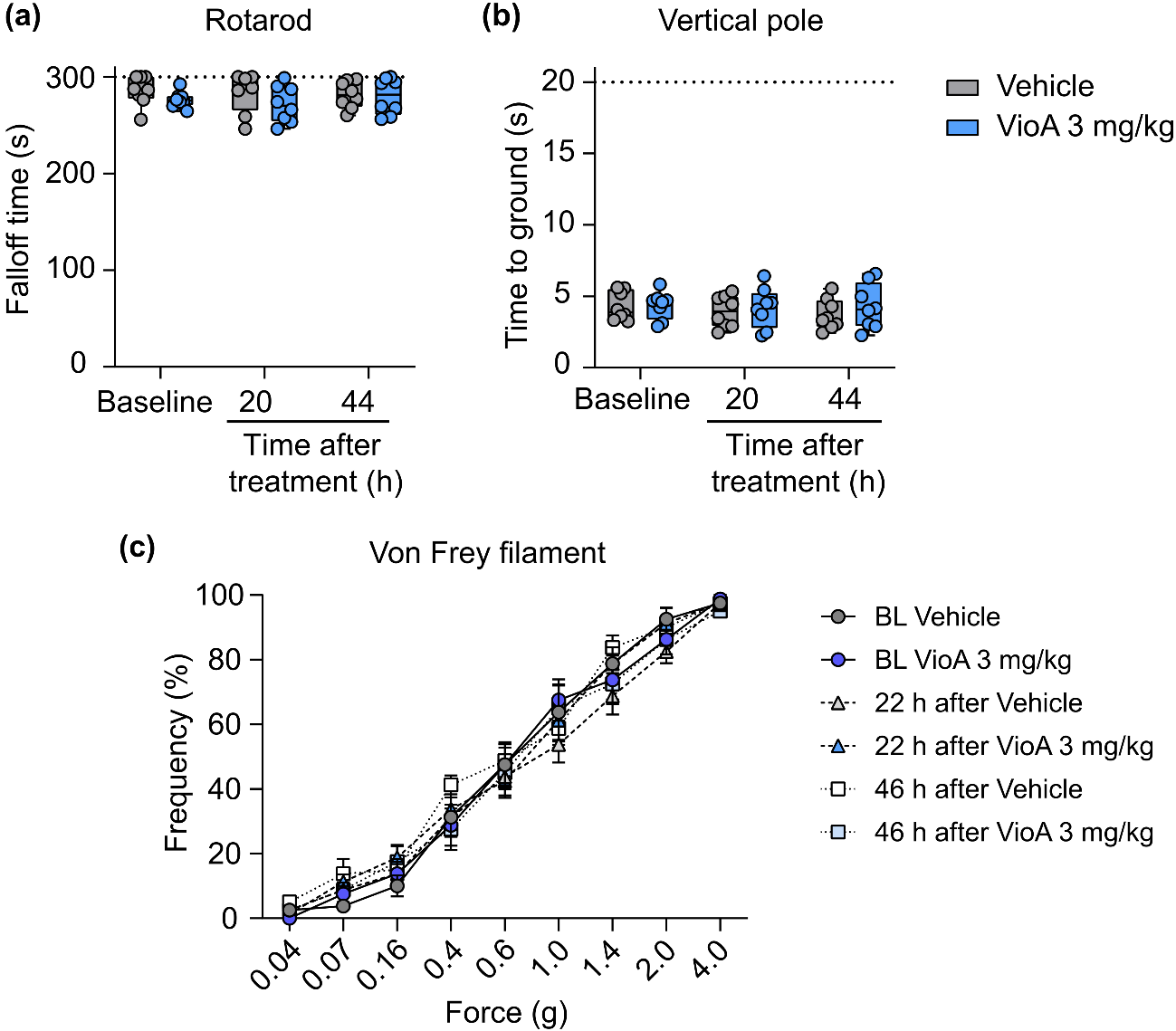
**

**FIGURE S1** Vioprolide A does not impair motor function and mechanical sensitivity. (a and b) Motor function. Vioprolide A (VioA; 3 mg/kg) or vehicle (1% DMSO in 0.9% NaCl) were subcutaneously (s.c.) administered into the neck area, and an accelerating rotarod test (a) followed by a vertical pole test (b) was performed 20 h and 44 h thereafter. Data show that vioprolide A did not affect the time spent on the rotarod or the vertical pole. Box-and-whisker plots represent maximum and minimum values, and the box shows the first, second (median), and third quartile values. Dotted lines indicate the maximum test time (cutoff time). n = 8 mice per group. (c) Percentage of paw withdrawals in response to von Frey filaments at different forces (ten trials per filament) in mice 22 h and 46 h after s.c. administration of vioprolide A or vehicle. BL: Baseline. Data are means ± SEM from n = 8 mice per group.
