# Supplementary material for: The mRNA Translation Inhibitor Vioprolide A Prevents Inflammatory Pain‐Like Behaviour With Limited Action on Already Established Pain‐Like Behaviour in Mice
Source: Eur J Pain. 2025 Aug 13;29(8):e70099. doi: 10.1002/ejp.70099 (PMC12345401; doi:10.1002/ejp.70099)
Supplement: Supplementary file 2 — Figure S2: NOP14 expression in dorsal root ganglia is not regulated by vioprolide A or zymosan. Sixteen hours after s.c. administration of vioprolide A (VioA; 0.3 mg/kg) or vehicle, mice received an intraplantar injection of zymosan (zym) into a hindpaw while control mice did not receive a zymosan injection. After 24 h animals were killed and L4–L5 DRGs were excised. (a) Representative Western blot of NOP14 (calculated molecular weight: 98 kDa) with DRG homogenates. α‐Tubulin (52 kDa) was used as loading control. (b) Densitometric analyses did not detect altered expression of NOP14 after vioprolide A treatment and/or intraplantar zymosan injection. n = 3 animals per group. [file EJP-29-0-s002.docx]

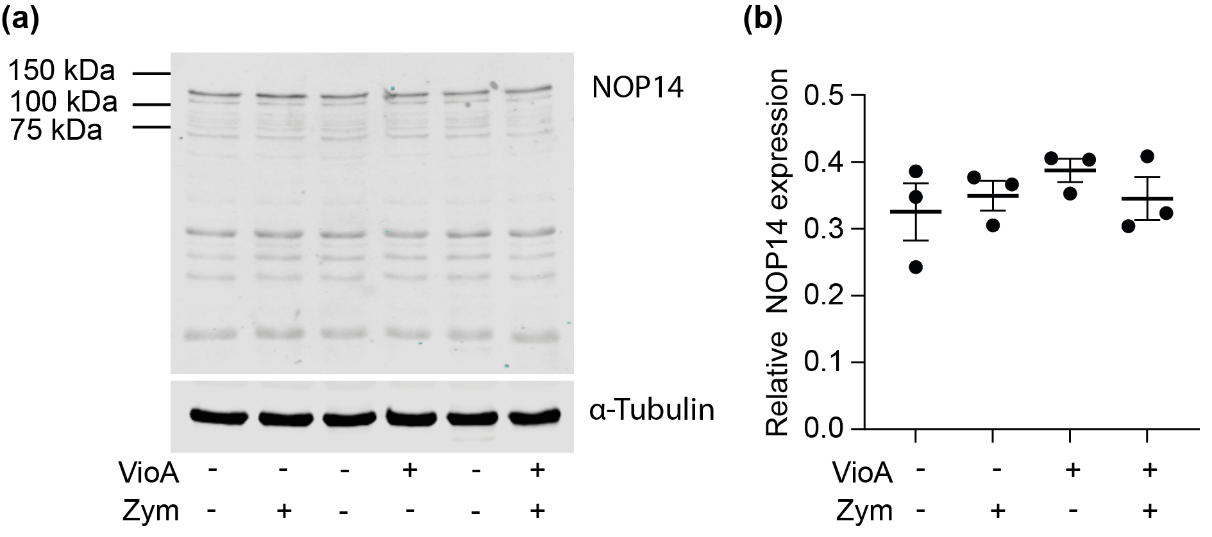


**FIGURE S2** NOP14 expression in dorsal root ganglia is not regulated by vioprolide A or zymosan. Sixteen hours after s.c. administration of vioprolide A (VioA; 0.3 mg/kg) or vehicle, mice received an intraplantar injection of zymosan (zym) into a hindpaw while control mice did not receive a zymosan injection. After 24 h animals were killed and L4–L5 DRGs were excised. (a) Representative Western blot of NOP14 (calculated molecular weight: 98 kDa) with DRG homogenates. α-Tubulin (52 kDa) was used as loading control. (b) Densitometric analyses did not detect altered expression of NOP14 after vioprolide A treatment and/or intraplantar zymosan injection. n = 3 animals per group.
